# Estimation of Serum Malondialdehyde (a Marker of Oxidative Stress) as a Predictive Biomarker for the Severity of Coronary Artery Disease (CAD) and Cardiovascular Outcomes

**DOI:** 10.7759/cureus.69756

**Published:** 2024-09-19

**Authors:** Asfandiyar, Noorul Hadi, Imran Ali Zaidi, Zeeshan Kamal, Ashraf, Rizwan Ullah Khan, Rumman, Mohammad Hashim Khan, Fatima Omair

**Affiliations:** 1 Cardiology, Medical Teaching Institution Mardan Medical Complex, Mardan, PAK; 2 Biochemistry, FMH College of Medicine and Dentistry, Lahore, PAK; 3 Biochemistry, Khyber Medical University Institute of Medical Sciences, Kohat, PAK; 4 Pathology, Pro-Gene Diagnostics and Research Laboratory, Mardan, PAK; 5 Pulmonology, Medical Teaching Institution Mardan Medical Complex, Mardan, PAK; 6 Cardiology, Khyber Medical University, Mardan, PAK; 7 Biochemistry, Pro-Gene Diagnostics and Research Laboratory, Mardan, PAK

**Keywords:** inflammation, oxidative stress, cardiovascular outcomes, malondialdehyde, coronary artery disease

## Abstract

Background

Coronary artery disease (CAD) is influenced by oxidative stress, which is a critical yet often overlooked factor in disease progression. While traditional biomarkers such as cholesterol levels and blood pressure are commonly used, they do not fully capture oxidative damage. Malondialdehyde (MDA), a byproduct of lipid peroxidation, offers additional insights into oxidative stress and CAD severity. Unlike conventional markers, such as low-density lipoprotein (LDL) cholesterol, which primarily reflects lipid levels, and high-sensitivity C-reactive protein (hs-CRP), which indicates inflammation, MDA directly measures oxidative damage. This makes MDA a potentially valuable complement to these traditional biomarkers, providing a more nuanced understanding of CAD risk. Despite its potential, the role of MDA in clinical assessments remains underexplored. This study aims to address this gap by evaluating MDA’s effectiveness as a complementary biomarker, enhancing the assessment of CAD risk and progression beyond what is provided by existing markers.

Objective

This study aims to assess serum MDA levels in relation to CAD severity to explore its potential as a non-invasive biomarker for disease progression and cardiovascular outcomes.

Methodology

This cross-sectional study was conducted at the Department of Cardiology, Mardan Medical Complex Teaching Hospital, Pakistan, from June 2023 to May 2024. Patients were divided into different groups with varying severity of CAD. The one-way ANOVA was used to assess differences among groups, and Pearson's correlation coefficient explored relationships between MDA and all study variables. Simple linear regression analyzed associations between MDA levels, patient groups, and other variables, controlling for covariates. MDA's potential as a predictive biomarker was assessed through ROC curve analysis, with statistical significance set at a p-value < 0.05.

Results

A total of 133 patients were included in the study, categorized based on CAD severity into mild (n=71), moderate (n=39), and severe (n=23) groups. Serum MDA levels significantly increased with the severity of CAD. Specifically, MDA levels were 116.61 ± 41.95 in the mild group, 253.45 ± 180.29 in the moderate group, and peaked at 459.91 ± 149.80 in the severe group. The differences in MDA levels among these groups were statistically significant (p < 0.01), supporting the association between higher MDA levels and increased CAD severity. Factors such as BMI, heart rate, blood pressure, and smoking status also significantly influenced MDA levels. Receiver operating characteristic (ROC) curve analysis demonstrated high diagnostic accuracy of MDA for assessing CAD severity, with area under the curve (AUC) values of 0.81 for moderate and 0.94 for severe CAD. Comorbid conditions such as diabetes mellitus were associated with elevated MDA levels.

Conclusion

Elevated serum MDA serves as a reliable, non-invasive biomarker for predicting CAD severity, with potential applications in clinical risk assessment and management strategies. By identifying patients with elevated oxidative stress early, clinicians can implement timely interventions, potentially slowing disease progression and improving outcomes.

## Introduction

Coronary artery disease (CAD), a leading cause of morbidity and mortality worldwide, poses a significant challenge to the healthcare system [1]. CAD is marked by the buildup of atherosclerotic plaques in the coronary arteries, which impairs blood flow and can lead to serious events, such as myocardial infarction and stroke [2]. Given the multifactorial nature of CAD, there is a crucial need for reliable biomarkers to aid in early detection, risk stratification, and effective management [3]. Among various oxidative stress markers, serum malondialdehyde (MDA) stands out as a promising biomarker for assessing the severity of CAD and predicting cardiovascular outcomes, yet its comparative advantage over other markers remains underexplored.

MDA, a byproduct of lipid peroxidation, reflects oxidative stress [4], which plays a role in the development of atherosclerosis and CAD. Oxidative stress results from an imbalance between reactive oxygen species (ROS) production and the body’s ability to detoxify these reactive intermediates or repair the damage [5]. In CAD, oxidative stress contributes to endothelial dysfunction, inflammation, plaque instability, and thrombosis, all critical in atherosclerosis progression [6]. Elevated serum MDA levels, indicative of oxidative stress, are hypothesized to correlate with CAD severity and predict cardiovascular events.

This study investigates the utility of serum MDA as a biomarker for CAD severity, examining whether elevated MDA levels correlate with advanced stages of CAD and adverse cardiovascular outcomes. This is particularly relevant as current methods for assessing CAD severity, such as angiography, are invasive and carry risks [7]. A non-invasive biomarker such as serum MDA has the potential to significantly enhance patient assessment and management.

The focus on MDA stems from its role in lipid peroxidation, central to atherogenesis. Lipid peroxidation produces various end products [8], including MDA, which can drive inflammatory processes within the arterial wall [9]. While the oxidative stress-atherosclerosis link is well established, the direct relationship between serum MDA levels and CAD has not been thoroughly explored. This study aims to deepen our understanding of MDA’s role in CAD, potentially leading to more targeted management and prevention strategies.

Current diagnostics for CAD, including angiography and traditional biomarkers such as cholesterol and troponin, are valuable but have limitations, especially in predicting disease severity and outcomes. Traditional biomarkers often do not fully reflect oxidative stress driving CAD progression, highlighting the need for new biomarkers such as MDA, which directly reflects oxidative stress through lipid peroxidation. MDA is stable and directly linked to oxidative stress, making it a promising marker for predicting CAD severity.

Previous studies support MDA’s potential as a CAD biomarker, showing a correlation between higher MDA levels and increased disease severity. This study further explores MDA’s predictive value and examines whether elevated serum MDA levels correlate with greater CAD severity and adverse outcomes. If validated, MDA could influence patient management by offering a non-invasive method to monitor oxidative stress, leading to earlier interventions and more targeted treatments. Additionally, MDA measurements could complement traditional diagnostics, providing a more comprehensive view of cardiovascular health.

## Materials and methods

Study design

This cross-sectional study was conducted at the Department of Cardiology, Mardan Medical Complex Teaching Hospital in Khyber Pakhtunkhwa, Pakistan, from June 2023 to May 2024. Patient data were collected using a convenience sampling method and classified into three groups based on the degree of stenosis in the major coronary arteries: (1) mild coronary artery disease (CAD) (less than 50% stenosis); (2) moderate CAD (50%-70% stenosis); and (3) severe CAD (more than 70% stenosis).

Additionally, clinical symptoms, such as chest pain and electrocardiogram (ECG) changes, were considered to further support the classification of disease severity. This combination of imaging and clinical assessment provides a robust framework for evaluating CAD severity.

Inclusion criteria

Inclusion criteria involved patients aged 18 and older with a confirmed diagnosis of CAD severity, based on specified stenosis criteria, who were on a stable CAD treatment regimen for three to six months.

Exclusion criteria

Patients under the age of 18, those with incomplete laboratory or evaluation data, and individuals with major comorbidities such as cancer, autoimmune diseases, stroke, pregnancy, lactation, alcohol or drug abuse, unstable psychiatric conditions, recent coronary artery bypass surgery or other significant surgeries within the past three months, as well as specific cardiovascular conditions such as heart failure, peripheral artery disease, or atrial fibrillation, were excluded from the study.

Sample size

The sample size was calculated based on key statistical considerations. The primary outcome measure is the difference in mean MDA levels among three groups of CAD severity (mild, moderate, and severe). Assuming a moderate effect size (Cohen’s d = 0.5), a significance level (α) of 0.05, and a power (1-β) of 0.80, the estimated sample size required per group is approximately 32 patients. This results in a total sample size of around 96 patients. To account for potential dropouts or missing data, the study would benefit from oversampling, bringing the recommended total to approximately 110-115 patients. The study ultimately included 133 patients, exceeding the calculated requirement, thus ensuring sufficient power to detect significant differences in MDA levels across the different CAD severity groups. Although the study did not experience a significant number of dropouts, a sensitivity analysis technique such as the bootstrap resampling technique was conducted to assess the robustness of the findings.

Data collection technique

Data, including demographic and clinical details, were collected through interviews and standardized questionnaires. For blood tests, the study collected both random and fasting blood samples from each patient to clarify the sampling process. Random blood samples were specifically obtained for measuring MDA levels, as fasting was not required for this biomarker. In contrast, fasting blood samples were collected for additional metabolic and biochemical tests, such as glucose and lipid profiles, to ensure measurement accuracy. MDA levels were measured using a commercially available enzyme-linked immunosorbent assay (ELISA) kit by ELK Biotechnology (Wuhan, China). To mitigate potential confounders, additional laboratory tests were conducted using Roche Cobas e411 and c111 analyzers, which included tests for troponin I, C-reactive protein, D-dimer, HbA1c, fasting and random blood glucose levels, lipid profiles (cholesterol, triglycerides, high-density and low-density lipoproteins), alanine transaminase, alkaline phosphatase, albumin, total bilirubin, creatinine, and blood urea. Hematological parameters, such as total leukocyte count and differential counts (neutrophils, lymphocytes, monocytes, eosinophils, basophils), were assessed using a Sysmex XN-330 hematological analyzer. Biomarkers, including total lipid, non-high-density lipoprotein (HDL), and very low-density lipoprotein (VLDL), were computed using specific formulas.

Statistical analysis

Continuous variables were summarized as mean and standard deviation, while categorical variables were reported as frequencies and percentages. Differences among the mild, moderate, and severe coronary artery disease (CAD) groups were analyzed using appropriate statistical tests tailored to the nature of the data. For continuous variables, the one-way analysis of variance (ANOVA) was employed to compare the mean values across the three CAD severity groups. For categorical variables, including demographic and clinical characteristics, the chi-square test was primarily used to assess differences in proportions across the groups. In cases where the expected frequencies were low, Fisher’s exact test was applied for more accurate statistical inference. The relationship between MDA and all studied variables was evaluated using Pearson's correlation coefficient, represented as standardized coefficients. Simple linear regression analysis was conducted to investigate associations between MDA and patient groups, along with other variables, while controlling for potential covariates. The discriminatory ability of MDA as a predictive biomarker was evaluated using receiver operating characteristic (ROC) curve analysis, with the area under the curve (AUC) calculated. A p-value of < 0.05 was considered statistically significant. All statistical analyses were performed using Statistical Product and Service Solutions (SPSS, version 29.9; IBM SPSS Statistics for Windows, Armonk, NY) software.

Ethical considerations

The study was conducted with approval from the Institutional Review Board of Bacha Khan Medical College, Pakistan (Reference No: 569/BKMC, Dated: 27/06/2023). All participants provided fully informed consent after being briefed about the study's purpose and procedures. Confidentiality of patient data was strictly maintained through anonymization; identifying information was removed and replaced with unique patient codes. Data were securely stored in encrypted systems, accessible only to authorized research personnel.

Participants were assured of their right to withdraw from the study at any point without any negative consequences. The study adhered to the ethical guidelines established by the Declaration of Helsinki and local regulatory standards. Additionally, any adverse events during the study were monitored and reported in compliance with institutional guidelines, although no significant adverse events were recorded.

## Results

In this study involving 133 patients with CAD, the participants were categorized into three groups based on disease severity: mild (53.38%), moderate (29.33%), and severe (17.29%). The gender distribution was nearly equal across these groups, with no significant difference in the severity levels. The average age of the patients was 55.29 years, with a slight increase observed in those with severe CAD. BMI averaged 29.15, and it was significantly higher in patients with more severe CAD. Vital signs, such as heart rate and blood pressure, showed a notable escalation with increasing CAD severity. Common symptoms, including chest pain, fatigue, and weakness, were more prevalent in severe cases. A history of myocardial infarction (MI) was also more frequent in patients with severe CAD. Additionally, electrocardiogram (ECG) changes, such as T-wave inversions and ST-segment depressions, were significantly higher in severe cases. Lifestyle factors, including smoking and physical activity, were similar across all groups; however, efficient sleep was significantly lower, and non-vegetarian diets were more common among patients with severe CAD. Comorbidities such as diabetes mellitus (DM) were present in 22.55% of the patients, with other conditions exhibiting varied prevalence (Table 1). 

**Table 1:** Demographic and Clinical Characteristics of Patients With Coronary Artery Disease (CAD) Based on Their Severity Levels BMI: Body Mass Index, SOB: Shortness of Breath, MI: Myocardial Infarction, DM: Diabetes Mellitus, COPD: Chronic Obstructive Pulmonary Disease, HBS: Hepatitis B Surface Antigen, HCV: Hepatitis C Virus, GERD: Gastroesophageal Reflux Disease, CKD: Chronic Kidney Disease, CLD: Chronic Liver Disease Data are presented as mean ± standard deviation for continuous variables and as frequency (percentage) for categorical variables. P values marked with * indicate results from ANOVA, ** represent results from the Chi-Square Test, and *** denote results obtained using Fisher's exact test. P values with **** representing no statistics were computed because the characteristic is either present in 100% or 0% or in only one group of the population. A p value of <0.05 was considered statistically significant.

Characteristic	All	Mild	Moderate	Severe	P value
Total patients	133 (100)	71 (53.38)	39 (29.33)	23 (17.29)	****
Male	76 (57.15)	41 (57.74)	21 (53.84)	14 (60.86)	0.85**
Female	57 (42.85)	30 (42.25)	18 (46.15)	9 (39.13)	
Age	55.29±7.56	54.69±7.09	55.49±7.78	56.83±8.66	0.49*
BMI	29.15±3.68	28.44±3.65	29.68±3.43	32.02±4.21	0.00*
Heart beat	91.38±12.36	83.34±8.48	97.18±8.50	106.39±7.68	0.00*
Systolic pressure	143.26±17.92	131.93±7.63	147.13±10.33	171.70±16.48	0.00*
Diastolic pressure	93.74±9.53	86.45±2.93	97.49±4.14	109.91±6.39	0.00*
Sign symptoms					
Asymptomatic	38 (28.57)	38 (53.53)	0 (0.00)	0 (0.00)	****
Chest pain	95 (71.43)	33 (46.48)	39 (100)	23 (100)	0.00***
Fatigue	60 (45.11)	6 (8.45)	31 (79.49)	23 (100)	0.00***
Weakness	68 (51.13)	6 (8.45)	39 (100)	23 (100)	0.00***
Nausea	42 (31.58)	0 (0.00)	19 (48.72)	23 (100)	0.00***
Sweating	54 (40.60)	3 (4.23)	28 (71.79)	23 (100)	0.00***
Pain in Other Parts of the Body	62 (46.62)	0 (0.00)	39 (100)	23 (100)	0.00***
SOB Class 1	42 (31.58)	42 (59.15)	0 (0.00)	0 (0.00)	0.00***
SOB Class 2	49 (36.84)	29 (40.85)	20 (51.28)	0 (0.00)	
SOB Class 3	33 (24.81)	0 (0.00)	19 (48.72)	14 (60.87)	
SOB Class 4	9 (6.77)	0 (0.00)	0 (0.00)	9 (39.13)	
Family history of CAD	71 (53.38)	37 (52.11)	21 (53.85)	13 (56.52)	0.41**
Evidence of previous Mi	24 (18.05)	0 (0.00)	11 (28.21)	13 (56.52)	0.00***
Current MI	14 (10.53)	0 (0.00)	5 (12.82)	9 (39.13)	0.00***
ECG Changes no changes	67 (50.38)	67 (94.37)	0 (0.00)	0 (0.00)	0.00***
ECG Changes T-wave inversions	29 (21.80)	3 (4.23)	15 (38.41)	11 (47.83)	
ECG Changes ST-segment depressions	33 (24.81)	1 (1.41)	24 (61.54)	8 (34.78)	
ECG Changes ST-segment elevation	4 (3.01)	0 (0.00)	0 (0.00)	4 (17.39)	
Smoking	67 (50.38)	37 (52.11)	21 (53.85)	9 (39.13)	0.49**
Physical Activity	66 (49.62)	36 (50.70)	21 (53.85)	9 (39.13)	0.52**
Sleeping Habit: Efficient Sleep	58 (43.60)	58 (81.69)	0 (0.00)	0 (0.00)	0.00**
Sleeping Habit: Fragmented Sleep	75 (56.39)	13 (18.30)	39 (100)	23 (100)	
Dietary Habits: Vegetarian	22 (16.54)	7 (9.86)	7 (17.95)	8 (34.78)	0.01**
Dietary Habits: Non-vegetarian	111 (83.46)	64 (90.14)	32 (82.05)	15 (65.22)	
Comorbidities					
DM	30 (22.55)	16 (22.53)	9 (23.07)	5 (21.73)	0.03***
Asthma	4 (3.00)	1 (1.40)	1 (2.56)	2 (8.69)	0.00***
COPD	4 (3.00)	2 (2.81)	1 (2.56)	1 (4.34)	0.00***
HBS	2 (1.50)	0 (0.00)	2 (5.12)	0 (0.00)	****
HCV	3 (2.25)	1 (1.40)	2 (5.12)	0 (0.00)	0.07***
GERD	4 (3.00)	3 (4.22)	0 (0.00)	1 (4.34)	0.04***
CKD	3 (2.25)	2 (2.81)	1 (2.56)	0 (0.00)	0.05***
CLD	2 (1.50)	1 (1.40)	0 (0.00)	1 (4.34)	0.00***

Serum MDA levels showed a significant increase corresponding to CAD severity, with levels reaching a peak of 459.91 ± 149.80 in the severe CAD group (p < 0.05). Significant differences in MDA levels were observed across all CAD severity stages. Troponin I levels, a marker of cardiac injury, were also substantially higher in severe CAD. D-dimer levels, associated with blood clot formation, notably increased in severe cases as well. CRP levels remained consistent across all groups. Prothrombin time (PT) and international normalized ratio (INR) both significantly increased with the severity of CAD. Blood glucose and hemoglobin A1C levels, however, remained stable across different severity levels. Sodium levels significantly increased with CAD severity, while other electrolytes remained unchanged. Lipid profiles revealed marked differences in severe CAD cases, with total lipids, cholesterol, and triglycerides being notably higher. Total leukocyte count (TLC) and its components, including neutrophils, lymphocytes, and monocytes, also showed significant variation. Kidney function indicators, such as Blood Urea and Creatinine, were elevated in severe CAD, and Uric acid levels also rose with increasing CAD severity. Liver function tests remained stable except for a slight variation in total bilirubin levels (Table 2).

**Table 2:** Biomarkers of Patients With Coronary Artery Disease (CAD) Based on Their Severity Levels Data are presented as frequency and percentage or as mean and standard deviation. MDA: Malondialdehyde, CtnI: Troponin I, CRP: C-Reactive Protein, PT: Prothrombin Time, INR: International Normalized Ratio, HBA1C: Hemoglobin A1C, RBG: Random Blood Glucose, FBG: Fasting Blood Glucose, HDL: High-Density Lipoprotein, Non-HDL: Non-High-Density Lipoprotein, LDL: Low-Density Lipoprotein, VLDL: Very-Low-Density Lipoprotein, TLC: Total Leukocyte Count, NEU: Neutrophils, LYM: Lymphocytes, MON: Monocytes, ESO: Eosinophils, BAS: Basophils, ALT: Alanine Transaminase, ALP: Alkaline Phosphatase P values with **** representing no statistics were computed because the characteristic is either present in 100% or 0% or in only one group of the population. The statistical test used is one-way ANOVA. P value <0.05 is statically significant.

Characteristic	All	Mild	Moderate	Severe	P value
Total patients	133 (100)	71 (53.38)	39 (29.33)	23 (17.29)	****
MDA	216.10±173.60	116.61±41.95	253.45±180.29	459.91±149.80	0.00
CtnI	1.11±3.05	0.12±0.07	1.54±3.16	3.44± 5.42	0.00
D-dimer	0.75±0.31	0.60±0.12	0.86±0.40	1.07±0.20	0.00
CRP	8.97±0.56	3.94±0.57	13.96±0.56	24.10±0.53	0.49
PT	44.43±19.61	29.35±6.76	55.08±12.00	72.91±11.48	0.00
INR	2.96±1.30	1.95±0.45	3.67±0.80	4.86±0.76	0.00
RBG	146.19±67.02	148.51±71.46	140.41±51.26	148.83±77.96	0.81
FBG	96.73±26.83	93.07±21.19	102.13±34.62	98.87±26.85	0.21
HBA1C	6.48±2.34	6.43±2.25	6.71±2.69	6.26±2.04	0.74
Sodium	144.63±5.46	141.55±4.12	148.56±4.00	147.48±5.55	0.00
Potassium	4.78±0.98	4.70±0.98	4.76±1.00	5.08±0.93	0.27
Chloride	89.97±4.80	89.78±4.95	90.44±4.72	89.77±4.60	0.77
Total lipids	753.32±85.71	727.74±60.08	719.77±63.76	889.19±46.47	0.00
Cholesterol	189.98±24.40	183.48±20.77	181.90±17.80	223.78±14.41	0.00
Triglycerides	259.76±44.45	248.94±33.70	244.56±39.15	318.91±33.93	0.00
HDL	40.16±5.18	39.80±5.63	40.28±4.71	41.04±4.52	0.04
Non-HDL	149.83±25.06	143.68±22.48	141.62±18.12	182.74±14.82	0.00
LDL	97.87±22.39	93.88±22.89	92.70±17.03	118.95±16.63	0.00
VLDL	51.95±8.89	49.789±6.74	48.91±7.83	63.78±6.78	0.00
TLC	13437±12364	11000±1622	16460±21954	15834±5918	0.043
NEU	8331±7666	6820±1006	10205±13611	9817±3669	0.041
LYM	3896±3585	3190±470	4773±6366	4591.96±1716	0.042
MON	671±618	550±81	823±109	791.72±295	0.041
ESO	335±309	275±40	411±548	395±147	0.041
BAS	201±185	165±24	246.91±329	237±88	0.43
Blood urea	49.00±31.16	40.64± 29.47	51.18±35.06	71.09±14.01	0.00
Creatinine	1.53±0.51	1.40±0.51	1.58±0.56	1.83±0.28	0.02
Uric acid	5.86±1.07	5.64±1.02	5.67±0.95	6.86±0.88	0.00
ALT	39.56±9.38	39.05±9.82	39.07±8.81	41.99±8.91	0.39
ALP	141.76±30.96	142.92±32.63	143.05±32.04	135.96±23.43	0.61
Albumin	4.44±0.31	4.45±0.31	4.38±0.31	4.5004±0.29	0.30
Total bilirubin	0.87±0.05	0.8923±0.05	0.86±0.06	0.8661±0.05	0.033

The factors influencing MDA levels in CAD patients were further analyzed, revealing that CAD severity was a major determinant, as indicated by significant coefficients. Specifically, CAD severity had an unstandardized coefficient of 165.24 and a standardized coefficient (beta) of 0.84. In contrast, age and gender had minimal impact on MDA levels. BMI was a significant factor, with a coefficient of 2.26 (beta=0.39). Heart rate and blood pressure also significantly influenced MDA levels, with coefficients of 6.92 (beta=0.52) for heart rate, 4.90 (beta=0.59), and 12.28 (beta=0.67) for systolic and diastolic pressures, respectively. Smoking emerged as another significant factor, with a coefficient of 19.77 (beta=0.37). The history of CAD had minimal influence on MDA levels. Significant ECG changes and evidence of previous MI, particularly current MI, had a substantial impact. Asymptomatic patients had lower MDA levels, while symptoms such as chest pain, fatigue, and weakness were associated with higher MDA levels (Table 3).

**Table 3:** Demographic and Clinical Characteristics Factors Influencing Malondialdehyde (MDA) Levels Dependent variable: Malondialdehyde (MDA. The statistical test used was simple linear regression. Unstandardized coefficients represent the actual change in the dependent variable (in this case, MDA levels) for a one-unit change in the independent variable, while standardized coefficients (beta values) reflect the relative importance of each predictor variable on a common scale, allowing for comparison across different units of measurement. P value <0.05 is statically significant.

Model	Unstandardized Coefficients		Standardized Coefficients	t value	P value
	B	Std. Error	Beta		
Diseases severity	165.24	13.69	0.84	12.06	0.00
Age	21.39	30.47	0.06	0.70	0.48
Gender	0.26	2.00	0.01	0.13	0.89
Body mass index	2.26	4.11	0.39	1.93	0.04
Heartbeat	6.92	1.06	0.52	6.49	0.00
Systolic pressure	4.90	0.72	0.59	6.73	0.00
Diastolic pressure	12.28	1.17	0.67	10.45	0.00
Smoking	19.77	12.76	0.37	3.01	0.04
Coronary artery disease history	-1.46	12.84	-0.010	-0.11	0.90
ECG changes	107.31	13.63	0.59	7.87	0.00
Evidence of previous MI	139.72	37.34	0.48	3.742	0.00
Current MI	448.08	29.84	0.79	15.01	0.00
Asymptomatic	-138.19	30.72	-0.42	-5.06	0.00
Chest pain	151.19	30.72	0.49	4.08	0.00
Fatigue	191.04	25.19	0.55	7.58	0.00
Weakness	182.45	25.11	0.58	7.66	0.00
Nausea	211.43	23.86	0.61	8.39	0.00
Sweating	204.54	24.35	0.59	8.40	0.00
Pain in other parts of the body	213.35	23.86	0.64	8.94	0.00
Shortness of breath	122.38	12.48	0.72	9.43	0.00

Various biomarkers influencing MDA levels in CAD patients were also identified, showing significant variations. Troponin I had a notable impact on MDA levels, with a coefficient of 42.08 (beta=0.84, t value=12.61, p=0.00). D-dimer similarly had a considerable effect, with a coefficient of 417.29 (beta=0.75, t value=13.45, p=0.00). Random blood glucose (RBG) had no significant impact, while PT and INR both significantly influenced MDA levels, with coefficients of 5.90 (beta=0.66, p=0.00) and 88.55 (beta=0.66, p=0.00), respectively. Fasting blood glucose (FBG) had a moderate effect. In lipid profiles, total lipids, cholesterol, and triglycerides significantly affected MDA levels, along with non-HDL and LDL. VLDL levels also had a notable effect. The total leukocyte count (TLC) and its components significantly influenced MDA levels. Blood urea and creatinine were also significant, with coefficients of 0.63 (beta=0.36) and 56.69 (beta=0.48), respectively. Uric acid significantly influenced MDA levels, whereas liver function tests such as alanine transaminase (ALT) and alkaline phosphatase (ALP) had minimal impact. Total bilirubin negatively influenced MDA levels, while albumin had no significant effect (Table 4).

**Table 4:** Different Biomarkers Influencing Malondialdehyde (MDA) Levels MDA: Malondialdehyde, CtnI: Troponin I, CRP: C-Reactive Protein, PT: Prothrombin Time, INR: International Normalized Ratio, RBG: Random Blood Glucose, FBG: Fasting Blood Glucose, HDL: High-Density Lipoprotein, NON-HDL: Non-high-Density Lipoprotein, LDL: Low-density Lipoprotein, VLDL: Very Low-Density Lipoprotein, TLC: Total Leukocyte Count, NEU: Neutrophils, LYM: Lymphocytes, MON: Monocytes, ESO: Eosinophils, BAS: Basophils, ALT: Alanine Transaminase, ALP: Alkaline Phosphatase Unstandardized coefficients represent the actual change in the dependent variable (in this case, MDA levels) for a one-unit change in the independent variable, while standardized coefficients (Beta values) reflect the relative importance of each predictor variable on a common scale, allowing for comparison across different units of measurement. Dependent variable: Malondialdehyde (MDA). The statistical test used was simple linear regression. P value <0.05 is statically significant.

Model	Unstandardized Coefficients		Standardized Coefficients	t value	P value
	B	Std. Error	Beta		
CtnI	42.08	3.33	0.84	12.61	0.00
D-dimer	417.29	32.08	0.75	13.45	0.00
CRP	1.81	0.45	0.41	0.15	0.04
PT	5.90	0.57	0.66	10.25	0.00
INR	88.55	8.61	0.66	10.25	0.00
RBG	0.02	0.09	0.02	0.27	0.78
FBG	0.68	0.23	0.35	2.96	0.04
HBA1C	19.89	2.71	0.47	1.43	0.15
Sodium	5.85	1.030	0.53	5.52	0.00
Potassium	10.68	6.47	0.21	1.65	0.10
Chloride	0.73	1.33	0.04	0.55	0.58
Total lipids	0.34	0.06	0.59	5.06	0.00
Cholesterol	0.99	0.24	0.43	3.99	0.00
Triglycerides	0.61	0.13	0.57	4.55	0.00
HDL	0.83	1.23	0.05	0.67	0.50
Non-HDL	0.90	0.24	0.41	3.71	0.00
LDL	0.65	0.28	0.53	2.32	0.02
VLDL	3.06	0.67	0.47	4.55	0.00
TLC	0.003	0.00	0.55	5.91	0.00
NEU	0.004	0.001	0.53	5.92	0.00
LYM	0.009	0.002	0.51	5.91	0.00
MON	0.055	0.009	0.51	5.91	0.00
ESO	0.10	0.018	0.52	5.91	0.00
BAS	0.18	0.031	0.51	5.90	0.00
Blood urea	0.63	0.19	0.36	3.18	0.02
Creatinine	56.69	11.96	0.48	3.06	0.03
Uric acid	19.35	5.72	0.41	3.37	0.01
ALT	-0.03	0.68	-0.004	-0.045	0.96
ALP	-0.09	0.20	-0.041	-0.47	0.63
Albumin	3.301	20.44	0.014	0.16	0.87
Total bilirubin	-270.26	106.73	-0.21	-2.53	0.13

**Figure 1:**
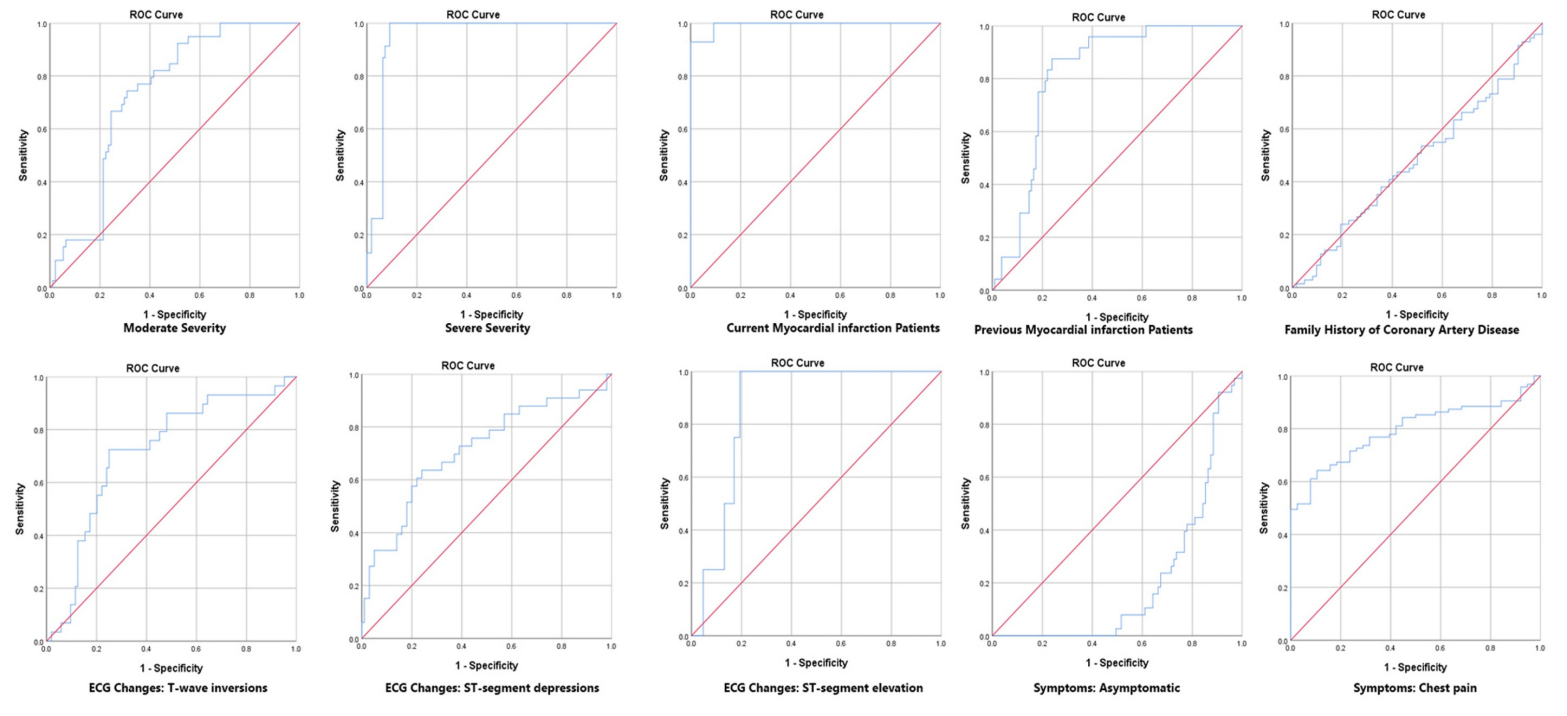
Comparative Analysis of Receiver Operating Characteristic (ROC) Curves for Various Diagnostic Metrics in Coronary Artery Disease Assessment

**Figure 2:**
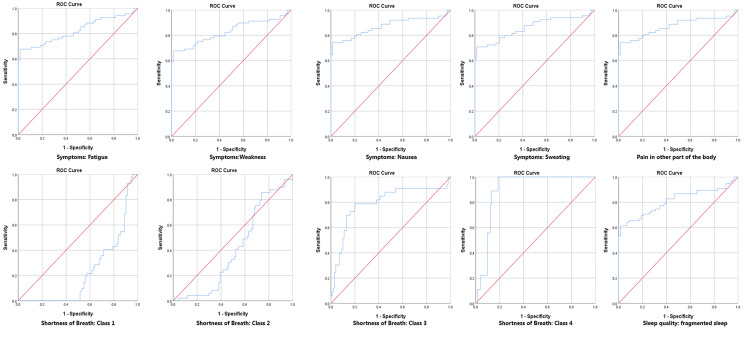
Comparative Analysis of ROC Curves for Various Diagnostic Metrics in Coronary Artery Disease Assessment

The diagnostic accuracy of MDA in assessing CAD severity was evaluated using the AUC. For disease severity, moderate CAD had an AUC of 0.81, and severe CAD had an AUC of 0.94, indicating high diagnostic accuracy. The current MI had an AUC of 0.99, reflecting excellent precision. In contrast, a history of CAD had an AUC of 0.47, indicating lower reliability. T-wave inversions and ST-segment depressions had AUCs of 0.78 and 0.82, respectively, while ST-segment elevation had an AUC of 0.86. Asymptomatic patients had a low AUC of 0.20. Symptoms such as chest pain and shortness of breath had high AUC values (0.80-0.87 for chest pain and 0.22-0.90 for shortness of breath), indicating good accuracy. Fragmented sleep quality also showed a strong correlation with CAD, with an AUC of 0.81 (Table 5, Figure 1, Figure 2).

**Table 5:** Diagnostic Accuracy Metrics for Diseases Severity, Cardiac Symptoms, and Conditions The statistical test used was receiver operator characteristics. AUC: Area under the curve. P value <0.05 is statically significant. Shortness of breath (SOB) was classified using the standard New York Heart Association (NYHA) functional classification, which assesses the severity of heart failure based on symptom severity and physical limitations. This classification is typically applied as follows: Class 1: No limitation of physical activity. Ordinary physical activity does not cause undue fatigue, palpitations, or shortness of breath. Class 2: Slight limitation of physical activity. Comfortable at rest, but ordinary physical activity results in fatigue, palpitations, or shortness of breath. Class 3: Marked limitation of physical activity. Comfortable at rest, but less than ordinary activity causes symptoms. Class 4: Unable to carry on any physical activity without discomfort. Symptoms of heart failure are present even at rest.

Characteristic	AUC	Std. Errors	P value	Asymptotic 95% Confidence Interval	
				Lower Bound	Upper Bound
Moderate severity	0.81	0.043	0.00	0.74	0.87
Severe severity	0.94	0.019	0.00	0.91	0.98
Current Myocardial Infarction	0.99	0.007	0.00	0.98	1.00
Previous Myocardial Infarction	0.82	0.038	0.00	0.74	0.89
Family history of coronary artery disease	0.47	0.50	0.64	0.37	0.57
T-wave inversions	0.78	0.05	0.00	0.72	0.83
ST-segment depressions	0.82	0.05	0.00	0.75	0.86
ST-segment elevation	0.86	0.39	0.01	0.78	0.94
Asymptomatic	0.20	0.38	0.83	0.13	0.28
Chest pain	0.80	0.38	0.00	0.72	0.86
Fatigue	0.82	0.038	0.00	0.75	0.89
Weakness	0.83	0.038	0.00	0.75	0.90
Nausea	0.87	0.035	0.00	0.80	0.94
Sweating	0.86	0.035	0.00	0.79	0.92
Pain in Other Parts of the Body	0.87	0.035	0.00	0.80	0.94
Shortness of breath					
Class 1	0.22	0.04	0.43	0.15	0.30
Class 2	0.41	0.49	0.08	0.31	0.50
Class 3	0.80	0.05	0.00	0.69	0.89
Class 4	0.90	0.02	0.00	0.84	0.95
Sleep quality fragmented sleep	0.81	0.03	0.00	0.73	0.88

**Figure 3:**
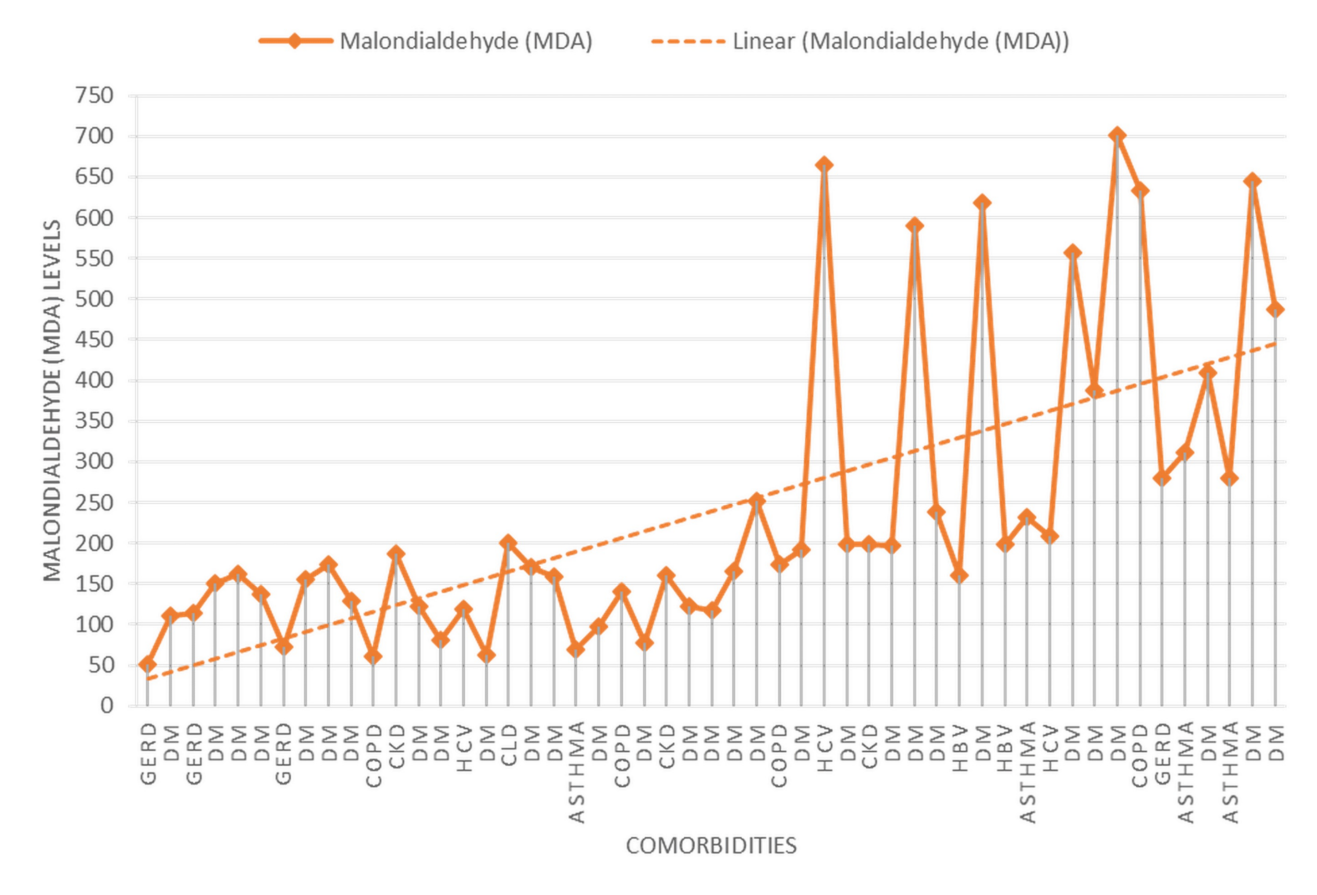
Trends and Variations in Malondialdehyde (MDA) Levels Across Different Commodities

Overall, MDA levels in patients with comorbidities averaged 239.0 ± 180. Among these patients, those with gastroesophageal reflux disease (GERD) exhibited the lowest MDA levels, while the highest levels were observed in patients with hepatitis C Virus (HCV) and diabetes mellitus (DM). Notably, one HCV patient with elevated MDA levels also had MI, indicating significant oxidative stress. Other conditions, such as chronic obstructive pulmonary disease (COPD), chronic kidney disease (CKD), chronic liver disease (CLD), hepatitis B Virus (HBV), and asthma, also showed elevated MDA levels, though not as high as that of DM (Figure 3).

**Figure 4:**
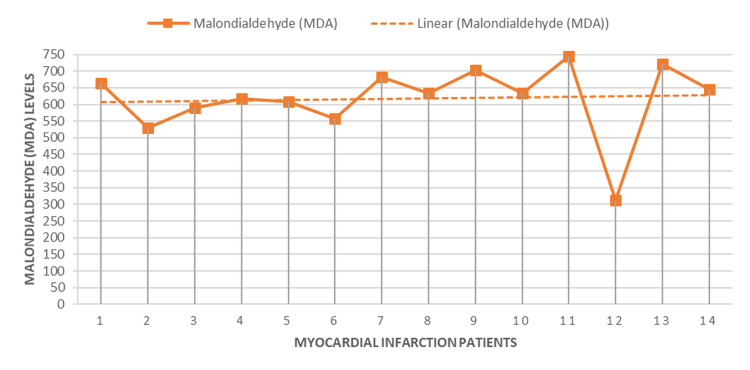
Trends and Variations in Malondialdehyde (MDA) Levels Across Different Myocardial Infarction Patients

Most MDA levels clustered around 617.14 ± 106.42, suggesting a relatively high level of oxidative stress in MI patients. However, there was one notable outlier where the MDA level sharply dropped below 300 units for a single patient before rising back to the previous range, indicating significant variability in oxidative stress levels among MI patients (Figure 4).

## Discussion

This study offers strong evidence supporting the utility of serum MDA as a reliable biomarker for evaluating the severity of CAD and its potential to predict adverse cardiovascular outcomes. A significant finding is the progressive increase in MDA levels corresponding with the severity of CAD. In patients with mild CAD, MDA levels averaged 116.61 ± 41.95, substantially increasing to 459.91 ± 149.80 in severe cases. This gradient of oxidative stress, as indicated by MDA levels, aligns with the theory that oxidative mechanisms play a crucial role in the progression of atherosclerosis and subsequent cardiovascular events [10]. The observed gradient suggests a direct relationship between the intensity of oxidative stress and the advancement of CAD. Oxidative stress is known to contribute to endothelial dysfunction, inflammation, and plaque instability, all key elements in the progression of CAD [11]. The increasing levels of MDA with CAD severity underscore its potential as a reliable marker for assessing the progression and prognosis of the disease. The significant variance in MDA levels across different severity stages in our study highlights the sensitivity of MDA as a biomarker, potentially surpassing traditional inflammatory markers such as CRP in detecting subtle changes in oxidative stress related to CAD.

Chen et al. found that serum MDA levels are closely associated with the severity of coronary heart disease, which aligns with our findings. In our study, MDA levels showed a marked increase with the severity of CAD, suggesting its potential as a biomarker for assessing disease progression [12]. Additionally, we observed significant elevations in troponin I (CtnI), D-dimer, and lipid profiles in patients with more severe CAD, consistent with existing literature, where elevated troponin I levels are associated with cardiac muscle damage, and D-dimer is linked to thrombotic processes [13-15].

Age and gender showed no significant impact on MDA levels. However, factors such as BMI, heart rate, blood pressure, and smoking status significantly influenced MDA levels, underscoring the multifactorial nature of CAD and its biomarkers [16]. Interestingly, CAD family history had minimal impact on MDA levels, suggesting that MDA reflects current oxidative stress rather than historical cardiac conditions. Our study’s observations on MDA levels in patients with comorbid conditions provided additional insights. Patients with conditions such as diabetes mellitus (DM) exhibited elevated MDA levels, indicating heightened oxidative stress in these conditions. This is consistent with research linking chronic diseases such as DM to increased oxidative stress; for instance, research has shown that patients with type 2 diabetes mellitus exhibit increased MDA levels, reflecting lipid peroxidation and oxidative stress [17].

The diagnostic accuracy of MDA for CAD severity, as reflected in the area under the curve (AUC) values, was noteworthy. The high AUC for severe CAD (0.94) indicates MDA’s potential as a reliable diagnostic tool [18]. In contrast, metrics such as a history of myocardial infarction (MI) showed lower AUC values, suggesting that MDA might offer more precision in assessing current disease severity compared to some traditional diagnostic measures. These findings highlight MDA’s role not only as a biomarker for severity assessment but also potentially in guiding treatment decisions. The ability to accurately assess CAD severity using a noninvasive biomarker such as MDA could significantly impact clinical practice, offering a more nuanced understanding of patient risk and prognosis.

Moreover, T-wave inversions and ST-segment depressions had AUCs of 0.78 and 0.82, respectively, while ST-segment elevation had an AUC of 0.86 for MDA. These findings suggest that MDA may offer greater precision in assessing current disease severity compared to some traditional diagnostic measures. Previous studies have demonstrated that ECG changes, including widespread ST-segment depression often associated with widespread inversion of the T wave, are indicative of severe CAD in non-ST elevation acute coronary syndrome, highlighting the significance of these ECG findings in CAD severity assessment [19]. Furthermore, the importance of isolated T-wave inversion and ST-segment depression in the electrocardiographic assessment of non-ST elevation acute coronary syndrome is well-supported in the literature, further underscoring the relevance of these ECG findings in CAD severity evaluation [20]. The association of ST-T segment abnormalities, such as T-wave inversion and ST-segment elevation or depression, with CAD highlights their potential significance in automated CAD detection [21].

Limitations

The conclusions of this study are subject to several limitations. First, the cross-sectional design provides data at a single point in time, which limits our ability to establish causal relationships or track changes over time. Implementing a longitudinal design could offer better insights into how MDA levels correlate with CAD progression and how changes in oxidative stress impact disease outcomes. Second, the limited sample size and single-center nature of the study may reduce the generalizability of our findings. Future research should involve larger, more diverse populations across multiple centers to validate these results and enhance their applicability. Third, the predominantly elderly patient cohort introduces potential bias, as advanced age is an independent risk factor for elevated MDA levels. The findings may not fully represent younger populations, and future studies should encompass a broader age range for a more comprehensive understanding of CAD across different demographics. Fourth, higher BMI is linked with increased oxidative stress and inflammation, which might confound the relationship between MDA levels and CAD severity. Although efforts were made to control for various covariates, the influence of BMI on MDA levels might still skew results. Future research should include more detailed stratification and analysis of BMI categories. Additionally, other demographic and clinical confounders, such as medication use, diet, exercise, and comorbidities such as hypertension and diabetes, could significantly impact MDA levels. Medications, such as statins and antihypertensives, and antioxidant supplements could alter oxidative stress markers, and detailed documentation and control of these factors are necessary. Furthermore, one limitation of the study is that potential seasonal variations and environmental factors, such as temperature and air pollution, which can influence oxidative stress levels, were not accounted for. These factors could cause fluctuations in MDA levels independent of CAD severity, potentially affecting the study's results. Future studies should consider controlling for environmental conditions or conducting research across different seasons to minimize these effects.

## Conclusions

The study highlights the significant role of MDA as a biomarker for CAD, showing a strong correlation between MDA levels and CAD severity. This correlation suggests that MDA can serve as an early indicator of disease progression and offer valuable diagnostic and prognostic insights alongside traditional cardiovascular risk factors. However, it is crucial to emphasize that MDA should be integrated into a composite biomarker panel rather than used as a standalone predictor, given its nonspecific nature as a marker of oxidative stress. While the potential for MDA levels to be modifiable through statin therapy and lifestyle changes is promising, further clinical trials and studies are needed to substantiate these claims and provide robust evidence for their effectiveness. Therefore, while MDA shows potential as a modifiable risk factor, its role in clinical practice should be considered cautiously, with a focus on integrating it into a broader diagnostic and therapeutic framework.
